# Thyroid Nodule in an Eighteen-Year-Old Man as the First Presentation of Acute Lymphoblastic Leukemia

**DOI:** 10.5812/ijem.17364

**Published:** 2014-07-01

**Authors:** Majid Valizadeh, Minoush Moghimi, Abdolamir Feizi, Farbod Radmand, Zahra Piri

**Affiliations:** 1 Zanjan Metabolic Disease Research Center, Zanjan, IR Iran; 2 Department of Pathology, Zanjan University of Medical Sciences, Zanjan, IR Iran; 3 Student Research Committee, Zanjan University of Medical Sciences, Zanjan, IR Iran

**Keywords:** Thyroid Nodule, Acute Lymphoblastic Leukemia, Extramedullary, Lymphoma

## Abstract

**Introduction:**

Acute lymphoblastic leukemia (ALL) is the most common malignancy of childhood. Patients with ALL commonly present with easy bruising and infections due to medullary involvement. The extra medullary involvements of ALL manifests as hepatosplenomegaly, lymphadenopathy, and testicular enlargement. Among extramedullary manifestations of the ALL, thyroid involvement is rare. Herein, we reported a case of ALL that manifested as a thyroid nodule.

**Case Presentation:**

An 18**-**year-old young man with a thyroid nodule presented without any other symptom or sign. The excisional biopsy of the nodule was planned by the surgeon. After two months of lost to follow-up, the patient returned with a complaint of continuous bleeding after a tooth extraction. Peripheral blood smear (PBS) study and bone marrow aspiration proposed ALL and the flow cytometry confirmed the diagnosis. The R-Hyper-CVAD induction chemotherapeutic regimen (rituximab in combination with cyclophosphamide, vincristine, doxorubicin, and dexamethasone) was used for treatment. Interestingly, thyroid sonography and Tc^99m^ scan showed resolution of the thyroid nodule after chemotherapy.

**Discussion:**

In this patient, poor interdisciplinary communication and the rarity of this manifestation led to a delayed diagnosis. Therefore, we insist on more careful clinical examinations, reassessment of unusual FNA reports, and closer communication between clinicians and pathologists in such cases. This approach would lead to accurate and earlier diagnosis and would prevent unnecessary interventions.

## 1. Introduction

Acute lymphoblastic leukemia (ALL) is the most common malignancy of childhood with the peak age of 2-6 years (1). In the United States, ALL affects 1-2 out of 100000 individuals aged 15 to 19 years of which long term survival is 30 to 40% (2). Patients with ALL often present with fatigue, fevers, nocturnal perspiration, easy bruising, and infections due to the bone marrow involvement. Hepatosplenomegaly, lymphadenopathy, CNS disorders, cutaneous infiltration, and testicular enlargement are the most common extramedullary involvement (3).

Thyroid functional disorders (e.g. hypothyroidism) as a result of leukemic infiltration, are such uncommon extramedullary presentation of ALL that declared in case reports. (4). Based on a case series study, most of the ALL induced thyroid functional disorders, occurred during off-therapy follow-up because of thyroid gland damage by chemotherapy; moreover, hyperthyrotropinemia was reported in some patients after ALL chemotherapy (5). Although one case of primary hypothyroidism reported due to leukemic infiltration of thyroid gland (6), herein, we reported a case of ALL that was detected with thyroid nodule as its first presentation.

## 2. Case Presentation

**Figure 1.**
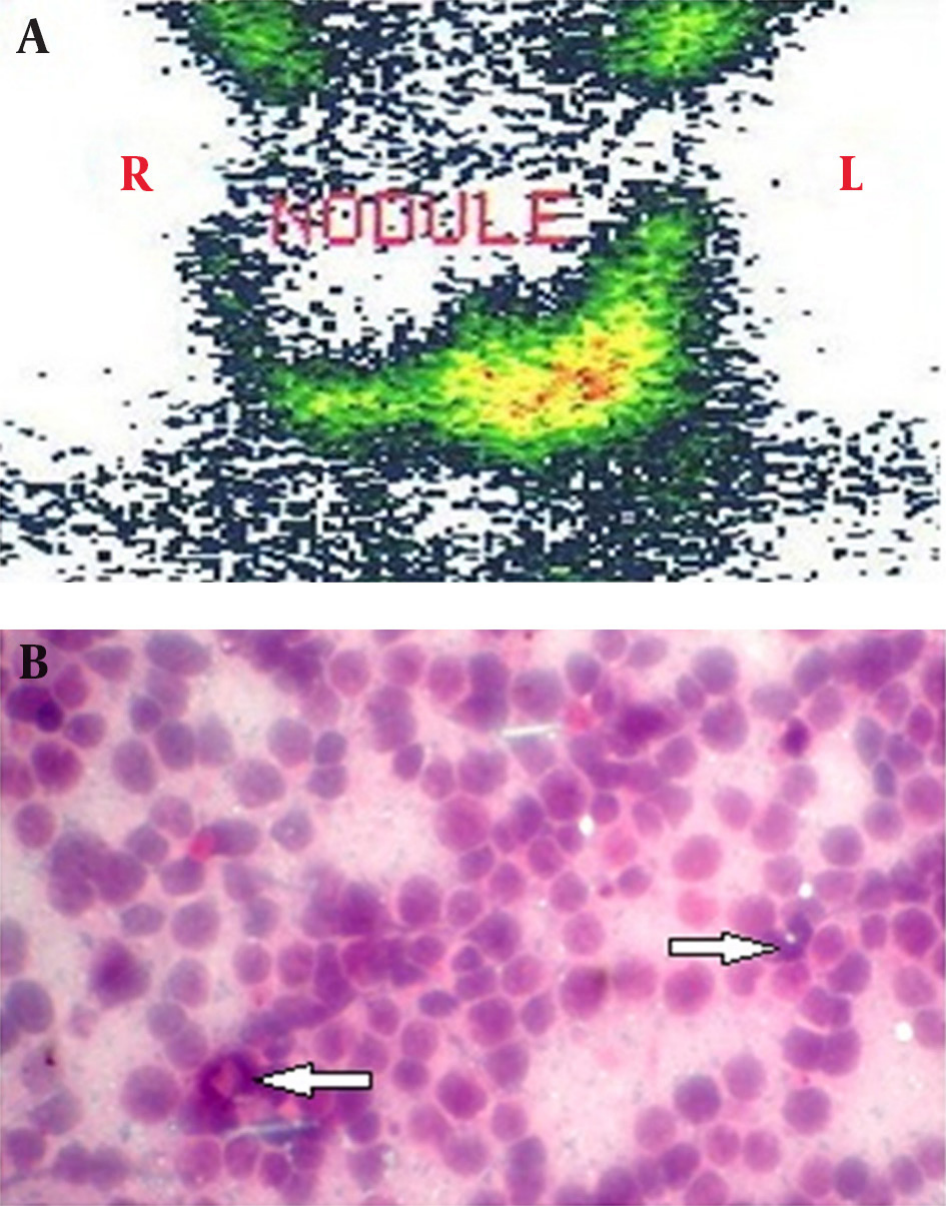
Thyroid Scan and Needle Aspiration A, thyroid scan with TC^99m^ before chemotherapy. B, Fine needle aspiration showing presence of lymphoid cells infiltration (Pap stain; magnification × 40).

The patient was an 18-year-old man with a recent detection of a thyroid nodule. The thyroid nodule was assessed by ultrasonography (USG) and Tc^99m^ scan. The thyroid USG reported a generalized enlargement with a heterogenic hypoechoic texture nodule with the size of 76 × 45 mm in the right lobe. Thyroid scan showed a cold nodule in the right lobe (Figure 1 A). Thyroid function test reported a normal level of thyroid stimulating hormone (TSH, 0.9 µIU/mL), free T4 (17 pmol/L), and free T_3_ (2.8 pmol/L). In addition, fine needle aspiration (FNA) reported presence of lymphoid cells infiltration suspicious of lymph node aspiration. Based on the suspicious FNA result, re-aspiration of the thyroid nodule performed that revealed a lymphoproliferative lesion (Figure 1 B).

A mild thrombocytopenia of about 123 ×10^3^ cell/μL was reported in complete blood count; however, as the patient had not any systemic symptoms, the clinician attributed it to the platelet aggregation. Based on this decision, he became candidate for excisional biopsy.

Unfortunately the patient was lost to follow-up for two months and he returned complaining of a continuous bleeding for two days after a tooth extraction. In addition, he expressed a history of constitutional symptoms like fever, chills, nocturnal perspiration, and cough in the last three weeks. During the physical examination, right cervical lymphadenopathy, enlarged and nodular thyroid, pale conjunctiva, splenomegaly, and left flank ecchymosis were detected. The patient was admitted to the hospital immediately.

In the next medical assessment, complete blood count revealed an Hb concentration of 10.5 g/dL, a WBC count of 12400 cell/μL, and a very low platelet count of 16 ×10^3^ cell/μL. The USG of the abdomen and pelvis revealed splenomegaly with the size of 185 mm.

PBS showed anisocytosis, hypochromia, decreased number of platelets and WBCs, and presence of around 5% blasts. Then, bone marrow was aspirated and examined that showed increased number of blasts and a significantly hypercellular marrow packed by immature cells with high nucleus to cytoplasm ratio and increased mitoses with minimal residual hematopoietic cells. The flow cytometry of bone marrow specimen showed positivity for HLA-DR, CD10, CD19, CD20, and CD45 as well as negativity for TdT. This report confirmed the diagnosis of pre-B-cell, Ph positive, chromosome negative, acute lymphoblastic leukemia.

**Figure 2.**
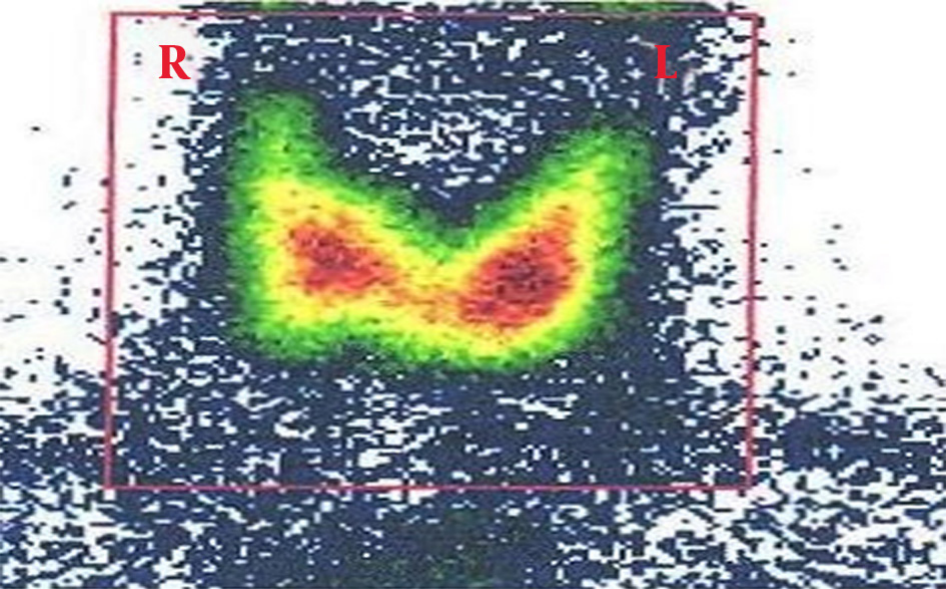
Thyroid Scan With Tc^99m^ After Chemotherapy Reporting Normal Radioactive Absorption

Based on the ALL diagnosis, the R-Hyper-CVAD induction chemotherapeutic regimen (rituximab in combination with cyclophosphamide, vincristine, doxorubicin, and dexamethasone) was started. Interestingly, thyroid examination after induction chemotherapy became normal. Thyroid USG indicated significant thyroid nodule regression with one hypoechoic texture nodule, 4 mm in size. Moreover, Tc^99m^ scan reported no hypoactive zone at the thyroid right lobe (Figure 2).

According to our experience it was so much difficult to perform an ultrasound guided FNA of a nodule 4 mm in size, therefore we relied on the thyroid USG and scan for the nodule regression. Beside the final flow cytometry of bone marrow aspiration reported ALL remission.

Thyroid function test reported the following values for TSH, T4, and anti-thyroid peroxidase (1.7 µIU/mL, 7.5 µg/dL, and 41.0 units/mL, respectively).

## 3. Discussion

This remarkable nodule regression after chemotherapy proved that ALL had a massive invasion to the thyroid before presenting with its usual manifestation. According to our knowledge, thyroid nodule as the first presentation of the ALL had not been reported before. It is worthy to note that most of the extramedullary thyroid involvements in the case reports manifested as hypothyroidism (5, 6). On the other hand, the most common lymphoproliferative disorder involving thyroid gland is lymphoma; however, it is an uncommon thyroid malignancy (7) and hence, thyroid leukemic infiltration has not any place in thyroid malignancies classification (8). In this case, there was a delay in accurate diagnosis caused by two reasons: firstly, rarity of this pathology, which is unfamiliar for clinicians; secondly, lack of interdisciplinary communication among different specialists while facing unusual cases in our setting.

Therefore, we emphasize on more careful clinical examinations and reassessment of strange FNA reports in encountering with an unusual case similar to our recent patient, especially when there is another hematologic finding. Moreover, we suggest closer communication between clinicians and pathologists as it would lead to accurate and earlier diagnosis and would prevent unnecessary interventions.
